# Effect of Breastfeeding and Preterm Births on the Severity of Lower Respiratory Tract Infections and Associated Risk of Hospitalization in Infants and Toddlers

**DOI:** 10.1177/2333794X221089762

**Published:** 2022-06-17

**Authors:** And Demir, Nihal Özdemir Karadas, Ulas Karadas

**Affiliations:** 1 University of Helsinki and Helsinki University Hospital, Helsinki, Finland; 2 Ege University Faculty of Medicine, Izmir, Turkey; 3 Dr. Behçet Uz Children’s Hospital, Izmir, Turkey

**Keywords:** breastfeeding, prematurity, preterm, pediatric, respiratory infection, hospitalization, infants, toddlers

## Abstract

We studied the effect of duration of breastfeeding and history of prematurity on the duration of hospitalization in infants with lower respiratory tract infections (LRTI) because these may reflect the severity of illness as well as sizable direct and indirect healthcare costs. One hundred twenty-five patients (49 girls, 76 boys; aged 1-24 months) were hospitalized for LRTI during a period of 102 days and studied prospectively. We found a significant difference (*P* = .045) between the durations of hospitalization of the 92 patients breastfed for at least six months, compared to the other group of 33 patients who were breastfed for less than six months. The durations of hospitalization among the groups with and without a history of prematurity were not statistically different (*P* = .78). A history of breastfeeding for more than six months had significant effect on the duration of hospitalization, but this was not true for children with a history of preterm birth.

## Introduction

Respiratory tract infections are a leading cause of morbidity and hospitalization among young children, particulary infants.^1^ Prospective cohort studies in industrialized countries revealed a prevalence of 3.4% to 32.1% for respiratory tract infectious diseases in infancy.^2^ Besides, the prevalance and mortality rates of lower respiratory infections, representing the relatively more serious disease spectrum, are much higher in developing countries than in industrialized countries; for instance, 15.4 versus 0.7 per 100,000 children under 5 years of age in Turkey and Finland, respectively.^3^ Indeed, lower respiratory tract infections (LRTI) are the leading cause of mortality in the 0-1 and 1-4 years old age groups in Turkey, the respective rates being 48.4% and 42.1%.^4^ These data show that lower respiratory infections in developing countries particularly in children under 5 years of age are a major public health problem leading to high mortality and morbidity.

Breastfeeding is well known to be among the protective factors for respiratory infections in infants. The protective role of breastfeeding against respiratory infections has been demonstrated in several studies on children living in developing countries.^5-7^ Also, a negative correlation between breastfeeding and hospitalization for respiratory infection was documented in studies performed in developed countries.^8^ However, the effect of breastfeeding on the prognosis of the disease or duration of hospitalization has not been documented so far.

Preterm infants, in particular, those born before 32 weeks gestation suffer even more significant respiratory morbidity because of lung immaturity at birth. Besides, they suffer also from relatively poor breastfeeding as they will not be strong enough to take enough nutrition by mouth to gain weight before 37 weeks. The more severe cases are diagnosed with bronchopulmonary dysplasia (BPD) based on oxygen requirement near term for corrected gestational age. However, infants born at less than 32 weeks who do not develop BPD and those born moderate to late preterm, that is, from 32 to 37 weeks gestation, also have an increased prevalence of respiratory symptoms and rehospitalization because of respiratory problems during their first year of life as well as a greater degree of respiratory symptoms at preschool age.^3,9,10^

Prior reviews from developed countries in regard to respiratory disease and its association with preterm births and breastfeeding have used illness episodes as their end point.^11,12^ Because both respiratory illness and infant feeding are evident on a broad continuum, investigation of this topic has been challenging and some published results may not correctly reflect the degree of cause-effect associations in regard to the reported benefits of breastfeeding in preventing severe disease. For this reason, we studied the effect of duration of breastfeeding or history of prematurity on the rate and duration of hospitalization for LRTI during infancy because these may reflect the severity of illness, and if so, they would be associated with sizable direct and indirect health care costs.

## Materials and Methods

### Patients and Samples

One hundred twenty-five patients (49 girls and 76 boys) aged between 1 and 24 months were hospitalized for LRTI according to clinical and radiological findings during a period of 102 days at Department of Pediatric Infectious Diseases, Dr. Behçet Uz Children’s Hospital University of Health Sciences, İzmir, Turkey were admitted and studied prospectively for several predefined parameters; that is, history of preterm birth, duration of breastfeeding, laboratory findings such blood culture and C-reactive protein (CRP), rate and duration of hospitalization, history of wheezing attacks or atopy prior to hospitalization. All patients with LRTI (acute bronchiolitis, tracheobronchitis, bronchopneumonia) were included in the study. Children with chronic diseases (severe malnutrition, congenital heart diseases, chronic respiratory system diseases, diabetes, chronic kidney disease, muscle and nervous system diseases, metabolic diseases) as well as patients with immunodeficiency were excluded from the study. The data were collected prospectively in the research with analytical characteristics. Demographic features were determined by taking detailed information about first and last name, age, gender, address, disease at the time of application. The following information was recorded as a history record for each patient: the date of the start of his complaints, attendance in nursery, presence of wheezing, number of attacks, the vaccination scheme and presence of chronic and allergic disease. Preterm birth has been defined as any birth before 37 weeks completed weeks of gestation. Also any history of disease during the neonatal period were also noted. Additionally, duration of breastfeeding was recorded in particular with reference to completion of six months on breast milk. The duration of stay in the hospital was recorded as short, moderate and long for stays shorter than 5, from 5 to 10 days and longer than 10 days, respectively. Finally, detailed information about family and ancestors was also recorded.

### Laboratory Studies

Hemogram was investigated in all patients. Blood culture was taken for the detection of possible bacterial agents and hourly sedimentation rates were determined in all the 125 cases. The number of WBC and the total eosinophil count were evaluated. Serum CRP was considered positive at 1 mg/dL and above, negative when the serum CRP level was below 5 mg/L, and serum CRP levels were determined to be mildly (5–10 mg/L), moderately (11–50 mg/L), (heavily 51–150 mg/L), and very heavily (>150 mg/L) elevated for respective values. Blood was drawn from each patient in order to identify the bacterial agent in cultures and to show possible bacteriemia. An anteroposterior x-ray, and if so required also a lateral one was taken from each case. Lung CT was taken only in patients if clinical and laboratory findings warranted so. An hourly red blood cell sedimentation rate higher than 20 mm/h and a white blood cell count below 5000/mL or above 14000/mL were regarded as pathological.

### Statistical Analyses

Parametric values were expressed as average ± standard deviation. Pearson’s chi-squared test was used for compares the distribution of counts for two or more groups using the same categorical variable. A *P*-value less than .05 was considered statistically significant.

### Ethical Approval and Informed Consent

Ethics committee approval (B.104.ISM.4356560/17) was obtained from Dr. Behçet Uz Children’s Hospital, University of Health Sciences, Izmir, Turkey before starting with the study. The study has complied with the World Medical Association Declaration of Helsinki regarding ethical conduct of research involving human subjects. During the study, all parents or custodians were notified with an information leaflet with details about the research study and all of them have provided written consent before the patients were admitted to the study.

## Results

The mean age of the patients admitted for hospitalization was 9.44 months (1-24 months) and the average length of stay was 3.9 days (minimum: 1 day, maximum: 13 days, SD: 2.4 days); duration of hospitalization was shorter than 5 days for 99 of the patients (79.2%), between 6 and 10 days for 21 of them (16.8%) and longer than 10 days for five of them (4.0%) (Table 1). There were no cases requiring cardiopulmonary resuscitation or intubation, nor was there any case of exitus.

**Table 1.** Effect of the Presence of a History of Preterm Birth on the Duration of Hospitalization (*P* = .78).

Duration of breastfeeding						
Effect on wheezing episodes and incidence of atopy						
	0-5 days		6-10 days		≥11 days	
	n (patients)	Percentage	n (patients)	Percentage	n (patients)	Percentage
History of preterm birth	(n = 99)		(n = 23)		(n = 3)	
Absent (n = 116)	92	92.93%	21	91.30%	3	100.00%
Present (n = 9)	7	7.07%	2	8.70%	0	0.00%

All cases were questioned in terms of premature birth. Nine (7.2%) of the 125 cases had premature birth history. When these nine cases were evaluated clinically, two of them presented with clinical findings for pneumonia, one of them for bronchiolitis, six of them for a combination of bronchiolitis and pneumonia. Lung X-ray was normal in five of these nine cases and the other four patients presented with the following findings: two of them with lung infiltrations, one of them with hyperlucent lungs of large volume and one of them with a combination of both findings. No statistical difference was detected between pulmonary graph findings of the patients with a history of prematurity when compared to the general population (*P* = .16).

Between the groups with and without history of prematurity, there was no statistical difference in regard to the rate of general findings for infection (*P* = .35), positive blood culture for bacteria (*P* = .40), positive viral serology (*P* = .86) and radiological findings (*P* = .98). The duration of hospitalization was also compared between the groups with and without history of premature birth. There was not any statistically significant difference between these two groups (*P* = .78) (Table 1).

Data from 125 patients over six months of age were analyzed in terms of length of breastfeeding periods and its effect on various parameters. Ninety-two patients (73.6%) received breast milk for at least six months and the remaining 33 patients (26.4%) for less than six months. There was no statistical difference between these two groups in regard to the growth percentiles (*P* = .29), radiological findings (*P* = .16), positive blood culture for bacteria (*P* = .23) or positive testing for viral serology (*P* = .79).

As for the rate of a positive CRP result among the patient group who were breastfed for at least six months and the other for shorter periods, a statistically significant difference was found (*P* = .009). Among the 125 patients who were tested for CRP, 33 of them were in the short breastfeeding group only eight of them (24.2%) tested negative. Among the remaining 92 patients who received breast milk for at least six months, 52 of them (56.5%) tested negative for CRP. In the patient group who were breastfed for less than six months, CRP positivity was at moderate levels in 22 cases (66.6%) (Table 2).

**Table 2.** Effects of Breastfeeding Time on Blood Culture, CRP Levels and Duration of Hospitalization.

	Breastfed < 6 months		Breastfed ≥ 6 months	
	n (patients)	Percentage	n (patients)	Percentage
	(n = 33)		(n = 92)	
Blood culture *P* = .234				
Negative	31	93.94	88	95.65
Contamination	1	3.03	4	4.35
Positive	1	3.03	0	0.00
CRP *P* = .009				
0-1 mg/dl	7	21.21	52	56.52
1-5 mg/dl	23	69.70	33	35.87
≥ 5.1 mg/dl	3	9.09	7	7.61
Duration of hospitalization *P* = .045				
0-5 days	22	66.67	77	83.70
6-10 days	8	24.24	13	14.13
≥ 11 days	3	9.09	2	2.17

We determined a statistically significant difference (*P* = .045) in regard to the duration of hospitalization period of the 92 patients breastfed for at least six months when compared to the other group of 33 patients who were breastfed for less than six months (Table 2).

We found no statistical difference between the two groups of patients who were breastfed for longer (at least six months) or shorter (less than six months) periods in terms of incidence of atopy (*P* = .69) (Table 3). Wheezing attacks were observed for more than three episodes in the group of five patients (15.2%) who were breastfed for less six months, and for three episodes or fewer in the group of 10 patients (10.9%) who were breastfed for more than six months; there was no statistical difference between the two groups in this regard (*P* = .55) (Table 3).

**Table 3.** Effect of Breastfeeding Time on Wheezing Episodes and Incidence of Atopy.

	Breastfed < 6 months		Breastfed ≥ 6 months	
	n (patients)	Percentage	n (patients)	Percentage
	(n = 33)		(n = 92)	
Wheezing episodes within the last 12 mo *P* = .55				
<3	28	84.85	82	89.13
≥3	5	15.15	10	10.87
Incidence of atopy *P* = .69				
Present	33	100.00	86	93.48
Absent	0	0.00	6	6.52

## Discussion

Studies performed in developing countries have reported that breastfeeding for long periods provides protection against infectious diseases in children.^13,14^ On the other hand, major differences have been observed between the results of similar studies on the same matter performed in developed countries.^5,6,12,15-18^ Regardless, breastmilk has been shown to include several protective factors such as immunoglobulins, lactoferrin, and lymphocytes, which in return has been associated with potential reductions in infant mortality also in developed countries.^12^

Some earlier studies have been presented with findings referring to breastfeeding as not providing any considerable protection against common infectious illnesses during the first year of life.^15^ On the other hand, some researchers have reported interruption of breastfeeding at short term as a risk factor for respiratory tract infections.^19^ Additionally, there have been some other reports advocating the benefits of breastfeeding for decreasing severity of possible respiratory viral infections, but not as a measure to prevent them.^18^ Our results confirm these findings from the aspect of severity of LRTI and duration of hospitalization in infants and toddlers up to 24 months of age.

Preterm infants in this study has not presented with any significantly longer periods of hospitalization compared to those born at term. In some earlier studies, Roggeri et al. had reported increased healthcare resource consumption and costs in preterm infants with bronchiolitis in the first year of life,^20^ and Backman et al. had reported long-lasting clinical consequences in infants with an acute and severe case of bronchiolitis in the first year of life.^21^ The results of these studies were based on comparisons among preterm infants with and without a history of LRTI during their first year of life. In this study, however, we have approached this issue by comparing the durations of hospitalization due to LRTI among preterm and normal term infants and found no significant difference in this regard.

As for limitations of this study, it should be noted that the association between formula feeding and the rate or duration of hospitalization and other indicators of severe infection were not considered in the current study design. In some earlier studies, parent observation was taken as one of the measures for defining the disease,^8,22,23^ for which required conditions were not available in this study.

As an additional finding of the study, breastfeeding was not found, however, to be a factor that would reduce the frequency and improve the symptoms of wheezing episodes or incidence of atopy. Results of this study have also revealed that presence of a history of preterm birth had no significant effect on the duration of hospitalization.

According to the results of this study, breastfeeding lasting longer than six months improved the prognosis by decreasing the severity of respiratory infections. Decrease in severity of disease was evident in statistically significant differences in several factors, such as CRP levels and duration of hospitalization. Indeed, infants who were breastfed for longer than six months recovered from respiratory infections after experiencing a milder infection with lower CRP levels and shorter periods of hospitalization. Evidence from an extensive birth cohort study revealed that approximately four out of 10 causes of all hospitalization cases is due to respiratory and gastrointestinal infections, and this rate was found to be even higher in infants up to 1 year of age.^24,25^

In conclusion, breastfeeding for at least six months or longer periods should be encouraged for improving the prognosis and decreasing the duration of hospitalization in children with LRTI up to 24 months of age. It remains to be studied further to observe whether similar benefits of breastfeeding exist also in older age groups.
